# Balloon pulmonary angioplasty can be an effective and safe therapeutic option in non-surgical elderly patients

**DOI:** 10.3389/fcvm.2022.1001518

**Published:** 2022-10-25

**Authors:** Maite Velázquez Martín, Nicolás Maneiro Melón, Agustín Albarrán González-Trevilla, Fernando Sarnago Cebada, Sergio Huertas Nieto, Alejandro Cruz-Utrilla, Williams Hinojosa, María Jesús López-Gude, Sergio Alonso Charterina, Yolanda Revilla Ostolaza, Ricardo José Aguilar Colindres, Fernando Arribas Ynsaurriaga, Pilar Escribano Subias

**Affiliations:** ^1^Cardiology Department, University Hospital 12 de Octubre, Madrid, Spain; ^2^Instituto de Investigación Sanitaria Hospital 12 de Octubre (imas12), Madrid, Spain; ^3^Centro de Investigación Biomédica en Red de Enfermedades Cardiovasculares (CIBERCV), Madrid, Spain; ^4^Cardiac Surgery Department, University Hospital 12 de Octubre, Madrid, Spain; ^5^Radiology Department, University Hospital 12 de Octubre, Madrid, Spain; ^6^Departamento y Facultad de Medicina, Universidad Complutense de Madrid (UCM), Madrid, Spain

**Keywords:** balloon pulmonary angioplasty (BPA), chronic thromboembolic pulmonary hypertension (CTEPH), pulmonary hypertension, survival, elderly patients, complications, hemodynamic benefits, reference center

## Abstract

**Background:**

Advanced age, frailty, and age-related comorbidities are the major causes of pulmonary endarterectomy disqualification in patients with chronic thromboembolic pulmonary hypertension (CTEPH). Balloon pulmonary angioplasty (BPA) is an attractive and less invasive therapy for elderly patients. However, information about the safety, procedure tolerance, and effectiveness of BPA in elderly patients is limited.

**Objective and methods:**

We aimed to analyze the safety, tolerance, and efficacy of BPA in CTEPH patients aged ≥70 years. This observational, descriptive, and retrospective series included consecutive patients aged ≥70 years, who underwent completed or interrupted BPA programs at a pulmonary hypertension reference center between May 2013 and May 2022.

**Results:**

We enrolled 155 patients in our institution's BPA program. Among these, 33 patients were aged ≥70 years (mean age, 76.4 years; women, 75.8%) and had finished or interrupted BPA programs. In this cohort, we performed 116 BPA procedures (average, 3.6 ± 1.8 sessions/patient). Among the 33 patients, 19 (57.6%) completed treatment for all lobes, while the BPA program was interrupted in the remaining 14 (42.4%). Among all 33 patients, BPA was associated with a significant reduction in mean pulmonary arterial pressure (39.2 ± 9.3 vs. 32.8 ± 8.8 mmHg; *p* < 0.001) and pulmonary vascular resistance (6.7 ± 3.1 vs. 4.4 ± 2.0 WU; *p* < 0.001), along with an improvement in the cardiac index (2.5 ± 0.6 vs. 2.8 ± 0.7 L/min/m^2^; *p* = 0.04) with significant reductions in the N-terminal prohormone of brain natriuretic peptide level (pre-BPA, 353 pg/mL [207–1,960 pg/mL] vs. post-BPA, 167 pg/mL [73–629 pg/mL]; *p* = 0.03). The patients' functional class improved, and pulmonary hypertension-targeting drug requirements were significantly reduced. The pulmonary injury appeared in 3.4% of the 116 procedures, of which 50% were of grade 2. No patient of ≥70 years had grade 5 pulmonary injury. One periprocedural mortality was recorded (3%), and the median follow-up period was 2.8 years. The survival rate of the entire cohort at 1 and 3 years was 90.5 and 82.8%, respectively.

**Conclusion:**

BPA is an effective and safe approach in patients aged ≥70 years. It significantly improves patients' functional class, hemodynamic, and biomarkers, and reduces their pulmonary hypertension-targeting medical therapy requirements. These successes were achieved even though a significant percentage of patients did not complete the therapy. The rates of procedural complications and periprocedural mortality were low. Survival at 1 and 3 years was good in comparison to that of younger patients undergoing BPA.

## Introduction

Chronic thromboembolic pulmonary hypertension (CTEPH) is caused by organized thrombi-induced obstruction and distal pulmonary vasculopathy. Surgical pulmonary endarterectomy (PEA) remains the method of choice for the treatment of CTEPH (1). However, not all patients are suitable candidates for surgery, either due to the lack of surgically accessible disease or unacceptable perioperative risk secondary to advanced age and comorbidities (2). Advanced age, frailty, and age-related comorbidities remain major causes of surgical disqualification. Balloon pulmonary angioplasty (BPA) has shown excellent results in patients with inoperable CTEPH, yielding improvements in functional class, hemodynamic status, and survival (3), and making it an attractive less invasive treatment option for elderly patients. However, since the poor functional class of these patients may have a multifactorial origin, they might benefit less from BPA. Moreover, since elderly people are usually frailer and often have more comorbidities, they could be prone to a higher rate of complications during BPA. Currently, there is little information regarding the safety, procedure tolerance, and effectiveness of BPA in elderly patients. Therefore, we aimed to analyze the safety, tolerance, and efficacy of BPA in patients aged ≥70 years at a pulmonary hypertension (PH) reference center.

## Methods

### Study patients

This observational, descriptive, and retrospective series included consecutive CTEPH patients aged ≥70 years, who were managed with BPA at our center between May 2013 and May 2022 and had finished BPA or interrupted the therapy for any reason. All of these patients were disqualified from PEA in weekly meetings by a multidisciplinary CTEPH team consisting of cardiac surgeons experienced in PEA, cardiologists specialized in the management of PH, radiologists with expertise in CTEPH imaging, and interventional cardiologists specialized in BPA. The decision to disqualify patients from PEA was based on their individual characteristics, including surgical accessibility, perioperative risk, and comorbidities. All patients were informed about their treatment options and the possible risks associated with BPA. All of them provided written consent and agreed to the processing of their data. This study complied with the Declaration of Helsinki for human research and was approved by the local ethics committee.

### Balloon pulmonary angioplasty procedure

**Figure 1:**
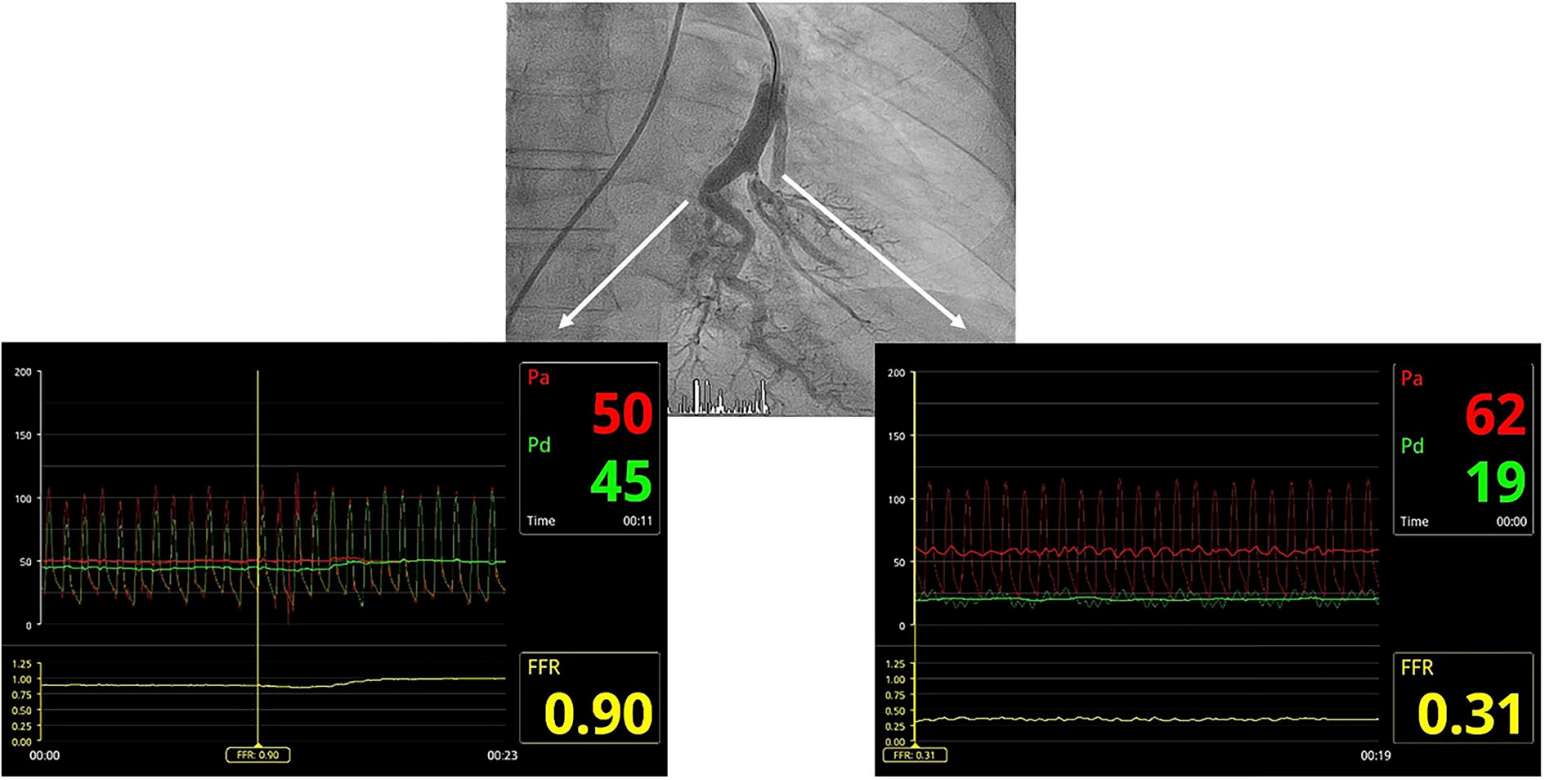
Utility of the pressure wire in clarifying angiographic images corresponding to arterial branch bends or true webs. The radiolucent image in the segmental branch on the left of the composition corresponds to an artery loop, with no significant pressure gradient between the guide catheter and the pressure wire distal to the lesion. The radiolucent image in the segmental branch on the right corresponds to a real web, with a significant pressure gradient between the guiding catheter and the pressure wire distal to the lesion, which is significantly damped.

**Figure 2:**
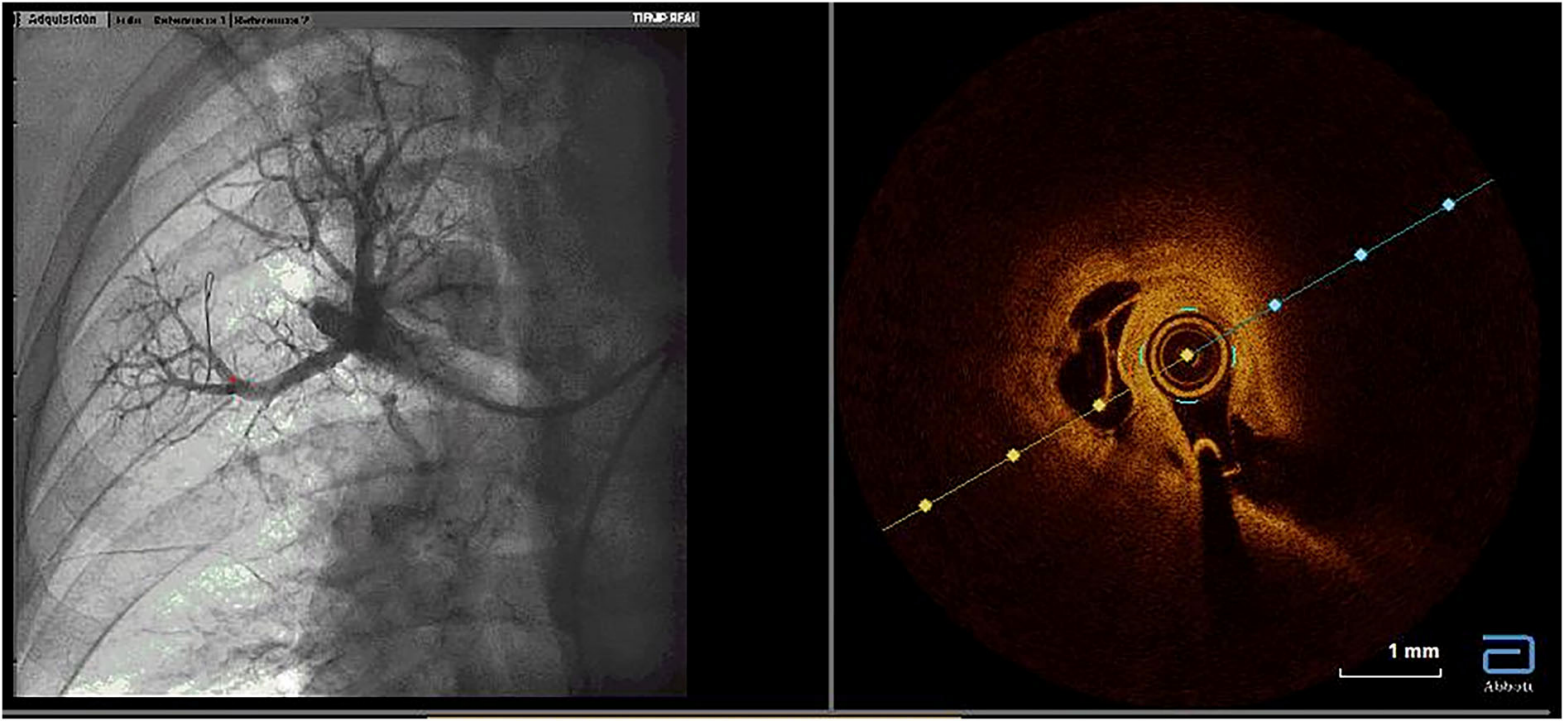
Utility of optical coherence tomography to confirm CTEPH organized thrombi inside pulmonary arteries. Organized thrombi inside one segmental branch of the upper right lobe. CTEPH, chronic thromboembolic pulmonary hypertension.

**Figure 3:**
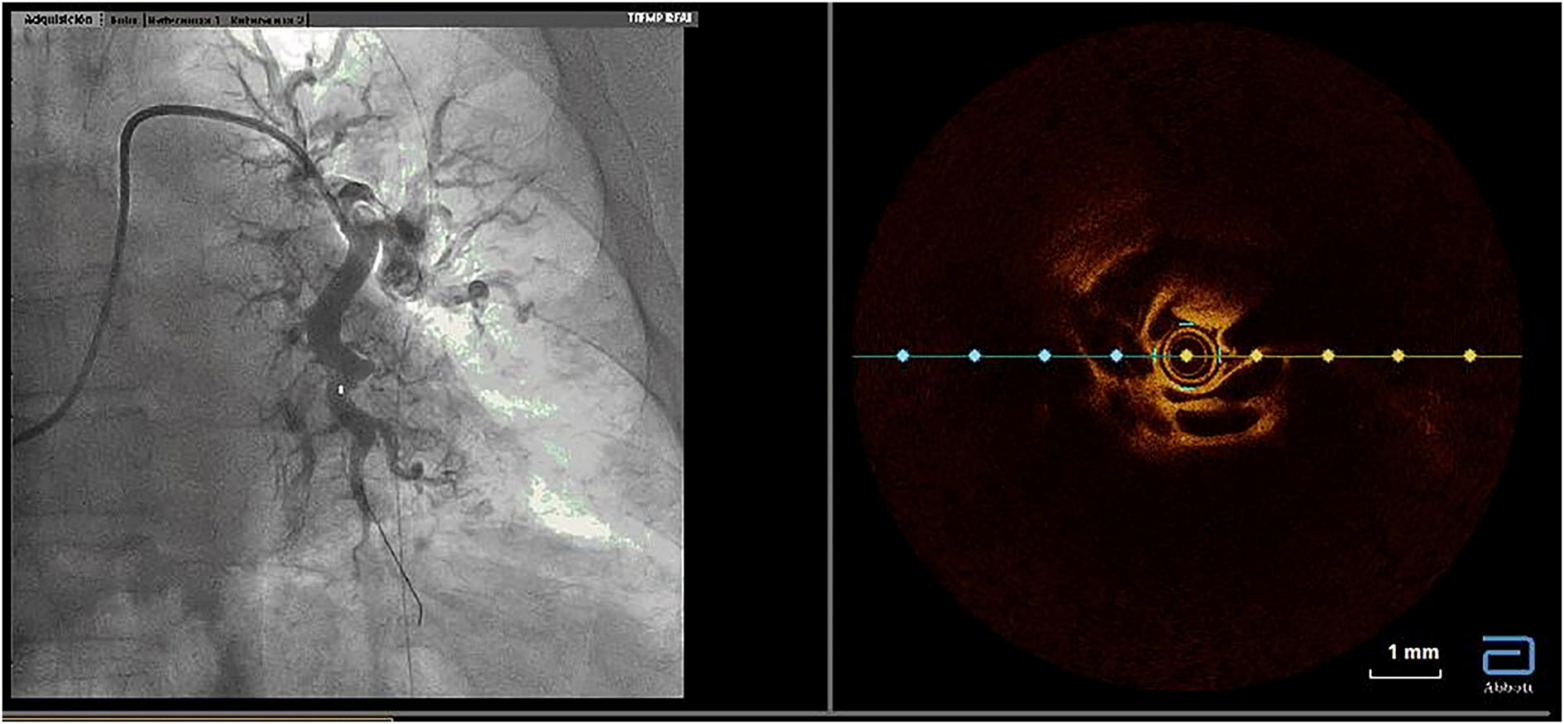
Utility of optical coherence tomography to confirm CTEPH organized thrombi inside pulmonary arteries. Organized thrombi inside the superior segmental branch of the left lower lobe. CTEPH, chronic thromboembolic pulmonary hypertension.

We performed BPA according to our previously described protocol (4). All procedures were performed by three senior interventional cardiologists with experience in the treatment of patients with PH and with substantial knowledge of the pulmonary vascular tree. Prior to BPA, warfarin administration was stopped and the patient received low molecular weight heparin (LMWH) at an anticoagulant dose for 48 h. The last scheduled dose of LMWH before the BPA procedure was not administered. The patient was awake and without sedation. The procedures were performed according to the standard refined technique (5). BPA therapy included the treatment of all amenable lesions in all lobes, with the purpose of normalization of hemodynamic parameters and functional class. The first procedure was focused on the lower lobes, since they are the bigger lobes. To avoid the development of reperfusion edema (RPE), we limited dilatations to 2 or 3 segmental branches of a single lobe per procedure as long as the mean pulmonary arterial pressure (mPAP) was >35 mmHg. Webs, bands, ring-like stenoses, and pouch lesions were treated. We used 0.014-inch polymeric, low-tip-load wires to cross the webs and bands. Dilatation began with undersized balloons, followed by balloons of increasing sizes up to a maximum of ≤ 80% of the vessel size. The intervention included the treatment of all treatable lesions in all lobes until normalization of hemodynamic parameters and functional class. Vessel size was estimated visually using selective pulmonary artery angiography. Intravascular ultrasound (IVUS), optical coherence tomography (OCT), and pressure wires were not used routinely and were employed only in cases involving doubts about lesion significance or to clarify the diagnosis of CTEPH (Figures 1–3) (6). Dilatation was considered effective when the distal flow improved, contrast uptake in the lung tissue distal to the treated area increased, and pulmonary venous return to the left atrium improved (Supplementary Videos 1, 2). After each procedure, we monitored patients for the development of RPE or pulmonary injury using chest radiographs obtained 8 h after each procedure. Patients remained hospitalized for 24–48 h to monitor complications.

### Definitions

We considered the BPA program to be completed when all balloon angioplasty-amenable lesions of all lobes had been dilated. The BPA program was considered to be interrupted when any of the following reasons caused therapy to be discontinued before achieving complete treatment of all amenable lesions: (1) personal decision to stop, (2) lack of functional and/or hemodynamic improvement (absence of improvement of at least 1 grade in the WHO functional class or a PVR reduction >20% after three BPA sessions), (3) inability of the patient to tolerate the procedures due to the duration and prolonged immobilization, or (4) severe complications. We analyzed the data from an intention-to-treat perspective, considering that patients had finished BPA therapy when they completed the program according to the protocol and when they discontinued the BPA program prematurely. Residual PH was defined as a final PVR > 416 din/s/cm^−5^ at least 6 months after the last BPA procedure (4). The appearance of lung opacities after the procedure in the treated lobe/s was considered to indicate reperfusion edema (RPE), which was recently renamed pulmonary injury. To classify RPE, a modified version of Inami's RPE classification was used, which also took into consideration the clinical impact of radiological findings (7) (Table 1). On the basis of angiographic findings, vascular injury during the procedure was classified as perforation, which showed contrast extravasation, or vascular dissection. Hemoptysis was classified as severe if it was accompanied by desaturation or if it required some intervention (balloon inflation or covered stent implantation) to stop it, and mild if it did not meet the criteria for severe hemoptysis. Periprocedural mortality was defined as the death of a patient that occurred 72 h following a BPA session or death during the index BPA hospital admission that was related to the BPA procedure. Acute renal failure was defined according to the KDIGO criteria (8).

**Table 1:** Reperfusion edema (or pulmonary injury) classification.

Grade 1	Absence of symptoms and absence of opacities on chest radiographs
Grade 2	Symptoms + drop in saturation + mild opacities on chest radiographs that improves with slightly increased oxygen supply
Grade 3	Symptoms + saturation drop + moderate opacities on chest radiographs, necessitating high concentrations of oxygen by mask to maintain arterial saturation at an optimal level
Grade 4	Symptoms + saturation drop + moderate-severe opacities on chest radiographs, necessitating noninvasive positive pressure ventilation and high oxygen concentrations to maintain arterial saturation at optimal level
Grade 5 (severe)	Symptoms + falling saturation + very severe opacities on chest radiographs, necessitating ECMO and/or mechanical ventilation

ECMO, Extracorporeal Membrane Oxygenator. Modified from Inami et al. (7).

### Complications

We recorded procedural complications of the 33 patients, including vascular injury, complications related to the puncture site, contrast allergy, development of pulmonary injury (RPE), and periprocedural mortality. Per-patient and per-procedure analysis of complications was performed.

### Clinical assessment during follow-up

All patients underwent standardized assessment prior to BPA. After finishing the BPA program, patients were evaluated at 6 months, 12 months, and yearly afterward. WHO functional class (FC), 6-min walking distance (6MWD), serum levels of the N-terminal prohormone of brain natriuretic peptide (NT-proBNP), and PH-targeting medical therapy were assessed, and right heart catheterization was performed at each scheduled visit. Mortality data were also collected during follow-up assessments.

### Statistical analysis

Statistical analysis was performed using the software STATA 14 (StataCorp, College Station, Texas, USA). The normal distribution of quantitative variables was evaluated using Shapiro–Wilk tests. Results with a normal distribution were displayed as mean ± standard deviation. All other variables were expressed as median (interquartile range; IQR). Differences in quantitative variables obtained at baseline and post-BPA were analyzed with Student's *t*-test for paired data or the Wilcoxon test. Categorical or ordinal variables were compared using the McNemar or sign test for paired data. A value of *p* < 0.05 was considered statistically significant. Survival analysis was performed using the Kaplan–Meier method and log-rank test.

## Results

**Figure 4:**
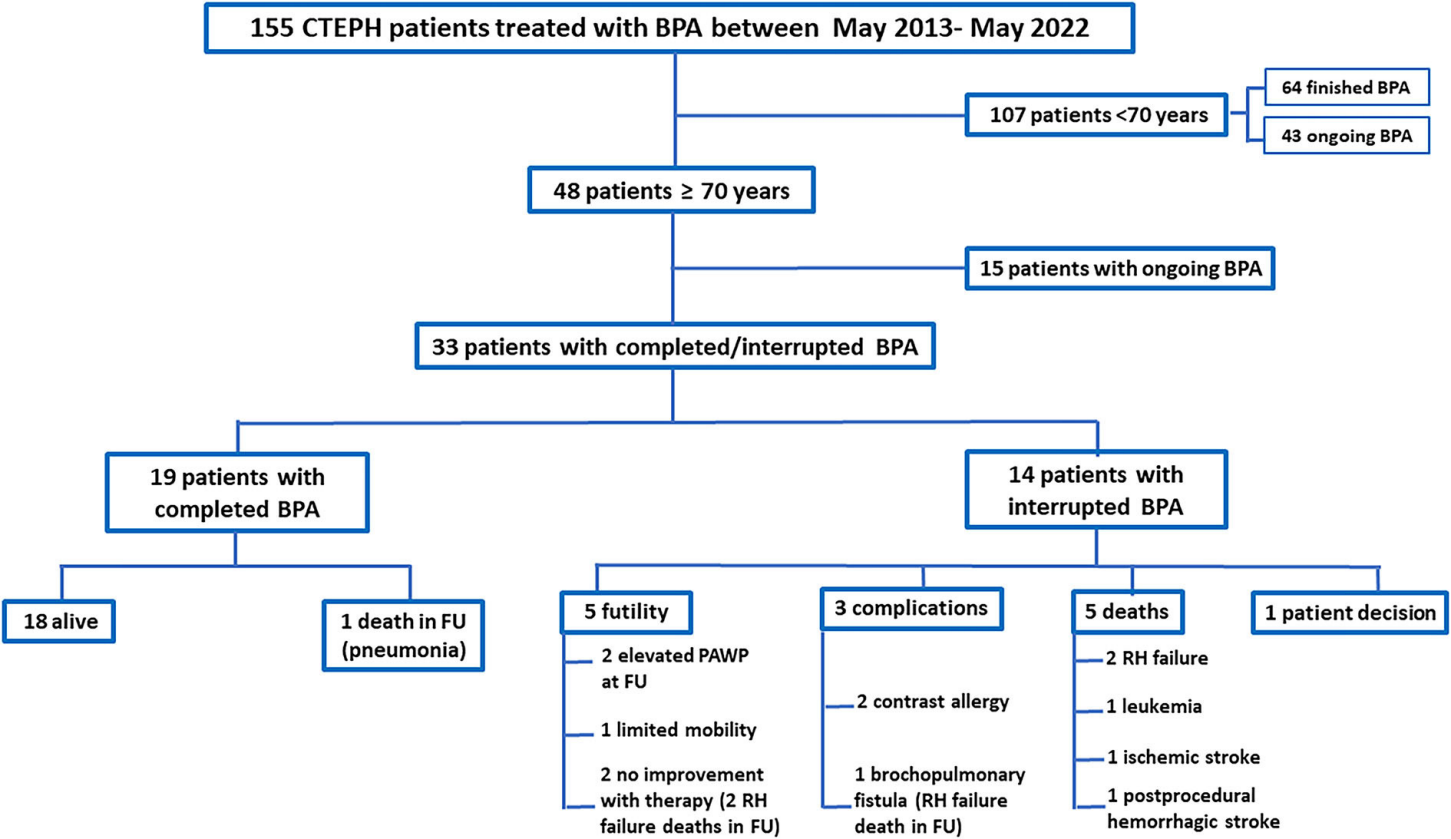
Patient flowchart. BPA, balloon pulmonary angioplasty; CTEPH, chronic thromboembolic pulmonary hypertension; FU, follow-up; PAWP, pulmonary artery wedge pressure; RH, right heart.

Between May 2013 and May 2022, a total of 155 patients were enrolled in our institution's BPA program. Forty-eight patients were aged 70 years or over, and 33 of them underwent a completed or interrupted BPA program (mean age, 76.4 years; women, 75.8%). We performed 116 BPA procedures in those 33 patients (average, 3.6 ± 1.8 sessions/patient). The indication for BPA was distal involvement in all but one of the patients (97%). Baseline demographic characteristics and procedural data are presented in Table 2, showing a predominance of women in our cohort. The patients' flowchart is shown in Figure 4. Of the 33 patients, 19 (57.6%) underwent treatment of all lobes and all lesions, while the BPA program was interrupted in the remaining 14 (42.4%) patients, including two patients who showed a severe allergic contrast, one patient who refused to continue with the BPA sessions after a serious complication (bronchopulmonary artery fistula) during the fifth procedure, one patient who refused to continue after his wife's death, five patients who died during the program period, and five patients who stopped receiving BPA therapy due to futility of the therapy in the opinion of their treating physicians. Table 3 shows the causes of death and futility considerations among the patients who did not complete BPA therapy. Those who completed the BPA program underwent an average of 4.1 ± 1.7 BPA sessions/patient, while those who did not complete the program underwent an average of 2.7 ± 1.6 BPA sessions/patient (*p* = 0.02). The median (IQR) BPA therapy length in those who completed therapy of all lobes was 371 days (287–545 days).

**Table 2:** Baseline demographic characteristics and procedural data (*n* = 33).

Age (years)	76.4 ± 3,4
Gender, women (%, *n*)	75.8 (25)
Body mass index (kg/m^2^)	28.7 ± 3.7
Thrombophilia (%, *n*)	12.1 (4)
Cancer history (%, *n*)	30.3 (10)
Hypertension (%, *n*)	66.7 (22)
Dyslipidemia (%, *n*)	30.3 (10)
Diabetes (%, *n*)	18.2 (6)
Atrial fibrillation (%, *n*)	12.1 (4)
Chronic coronary syndrome (%, *n*)	3.0 (1)
Asthma/COPD (%, *n*)	21.2 (7)
Smoking history (%, *n*)	15.2 (5)
Moderate-Severe renal insufficiency (%, *n*)	6.1 (2)
Hypothyroidism (%, *n*)	21.2 (7)
Disease anatomic site (%, *n*)	
•Central	3.0 (1)
•Peripheral	97.0 (32)
•Post-PEA	0
ERA (%, *n*)	33.3 (11)
PDEI (%, *n*)	30.3 (10)
Prostaglandins analogs (%, n)	21.2 (7)
Riociguat (%, *n*)	60.6 (20)
Diuretics (%, *n*)	69.7 (23)
O_2_ (%, *n*)	75.8 (25)
Number of BPA procedures/patient	3.6 ± 1.8
Fluoroscopy (min)	36.2 (28.4–46.5)
Contrast dose (ml)	299.6 ±−98.4
Procedure time (min)	118.9 ±−36.3

BPA, balloon pulmonary angioplasty; COPD, Chronic obstructive pulmonary disease; ERA, endothelin receptor antagonist; PEA, pulmonary endarterectomy; PDE5I, phosphodiesterase inhibitor; PH, pulmonary hypertension. Variables are expressed as percentages or mean ± SD.

**Table 3:** Causes of death, interruption, and futility considerations in the group with BPA therapy interruption.

	**Cause of therapy interruption during active BPA program**	**Number of BPA sessions before interruption**	**Comorbidities**	**Status during follow-up and cause of death**	**Age at death/stop (years)**	**PH treatment at death/stop**
**Death during active BPA program**						
· Patient 1	Hemorrhagic stroke 96 h after BPA	1	HT, cancer, COPD	Death related to BPA procedure	77.4	Prostaglandin + ERA + Riociguat
· Patient 2	Ischemic stroke	3	None	Death 1 year after last BPA	73.5	ERA
· Patient 3	Leukemia	6	HT, cancer	Death 6 months after last BPA	75	None
· Patient 4	Right heart failure progression	1	HT	Death 3 months after BPA session	72.5	Riociguat
· Patient 5	Right heart failure progression	2	HT	Death 10 months after last BPA session	74	Prostaglandin +ERA + Riociguat
**BPA program interruption due to futility**						
· Patient 1	Severely reduced mobility due to osteoarthritis	2	HT, DM, CKD, AF	Alive	82.4	ERA + PDEI
· Patient 2	No hemodynamic improvement after 3 BPA sessions	3	HT, DM, DL, AF	Death due to right heart failure progression 5 years after last BPA	84.4	Prostaglandin + ERA + Riociguat
· Patient 3	No hemodynamic improvement after 3 BPA sessions	3	CKD	Death due to right heart failure progression 19 months after last BPA	79.8	ERA + Riociguat
· Patient 4	Elevated PAWP at follow-up while in active program and poor clinical improvement	5	HT, DL, AF, cancer, COPD	Alive	77.4	Selexipag + Riociguat
· Patient 5	Elevated PAWP at follow-up while in active program and poor clinical improvement	2	HT, cancer	Alive	83.9	None
**BPA program interruption due to complication/personal decision**						
· Patient 1	Bronchopulmonary artery fistula	5	HT	Death due to right heart failure progression 2 years after BPA complication	81.2	Riociguat
· Patient 2	Serious contrast allergic reaction	2	HT, DM, smoking, cancer	Alive	74.2	Riociguat
· Patient 3	Serious contrast allergic reaction	1	Cancer	Alive	75.6	Riociguat
· Patient 4	Personal decision	2	Smoking	Alive	77.3	Riociguat

AF, atrial fibrillation; BPA, balloon pulmonary angioplasty; CKD, chronic kidney disease; COPD, chronic pulmonary obstructive disease; DM, diabetes mellitus; DL, dyslipidemia; ERA, endothelin receptor antagonist; HT, arterial hypertension; PAWP, pulmonary arterial wedge pressure; PDEI, phosphodiesterase inhibitor.

### Hemodynamic and clinical variables pre- and post-PABP

In a combined assessment of all 33 patients, BPA therapy was associated with a significant reduction in mPAP (mean reduction, 6.4 mmHg; 95% CI, 3.4–9.3; *p* < 0.001) and PVR (mean reduction, 2.3 UW; 95% CI, 1.4–3.3; *p* < 0.001) and an improvement in the cardiac index (mean increase, 0.29 L/min/m^2^; 95% CI, 0.02–0.55; *p* = 0.04). These patients also showed significant reductions in NT-proBNP levels (pre-BPA, 353 pg/mL [207–1,960 pg/mL] vs. post-BPA, 167 pg/mL [73–629 pg/mL]; *p* = 0.03) and in PH-targeting medical therapy requirements (mean reduction, 0.39 drugs; 95% CI, 0.11–0.67; *p* = 0.01). The patient's functional class also improved significantly. However, no significant differences were observed in the 6MWD (Table 4). Residual PH was present in 27.7% of the patients at follow-up.

**Table 4:** Hemodynamic and clinical changes after BPA in 33 patients aged ≥70 years.

**Variables**	**Pre-BPA**	**Post-BPA**	***p***
RAP (mmHg)	7.4 ± 3.3	7.4 ± 3.4	1
mPAP (mmHg)	39.2 ± 9.3	32.8 ± 8.8	**< 0.001**
CI (l/m^2^)	2.5 ± 0.6	2.8 ± 0.7	**0.04**
PVR (Wood units)	6.7 ± 3.1	4.4 ± 2.0	**< 0.001**
Peripheral O_2_ saturation (%)	93.0 ± 5.1	95.5 ± 2.8	**0.01**
Venous mixed O_2_ saturation (%)	65.2 ± 8.0	67.4 ± 5.8	0.13
6MW test (m)	325 (231–390)	351.5 (240–387)	0.23
NT-proBNP (pg/L)	353 (207–1690)	167 (73–629)	**0.03**
Number of PH-specific drugs	1.5 ± 0.8	1.1 ± 0.9	**0.01**
Number of PH-specific drugs (%)			
0	6.1	27.3	
1	51.5	48.5	
2	33.3	15.2	
3	9.1	9.1	**0.02**
WHO functional class (%)			
1	0	9.1	
2	21.2	63.6	
3	72.7	24.2	
4	6.1	3.0	**< 0.001**

6MW, 6-min walk; BPA, balloon pulmonary angioplasty; CI, cardiac index; mPAP, mean pulmonary arterial pressure; PH, pulmonary hypertension; PVR, pulmonary vascular resistance; RAP, right atrial pressure; WHO, World Health Organization; Pre-BPA, Pre-balloon pulmonary angioplasty; Post-BPA, Post-balloon pulmonary angioplasty; p, statistical significance.

### Complications

**Figure 5:**
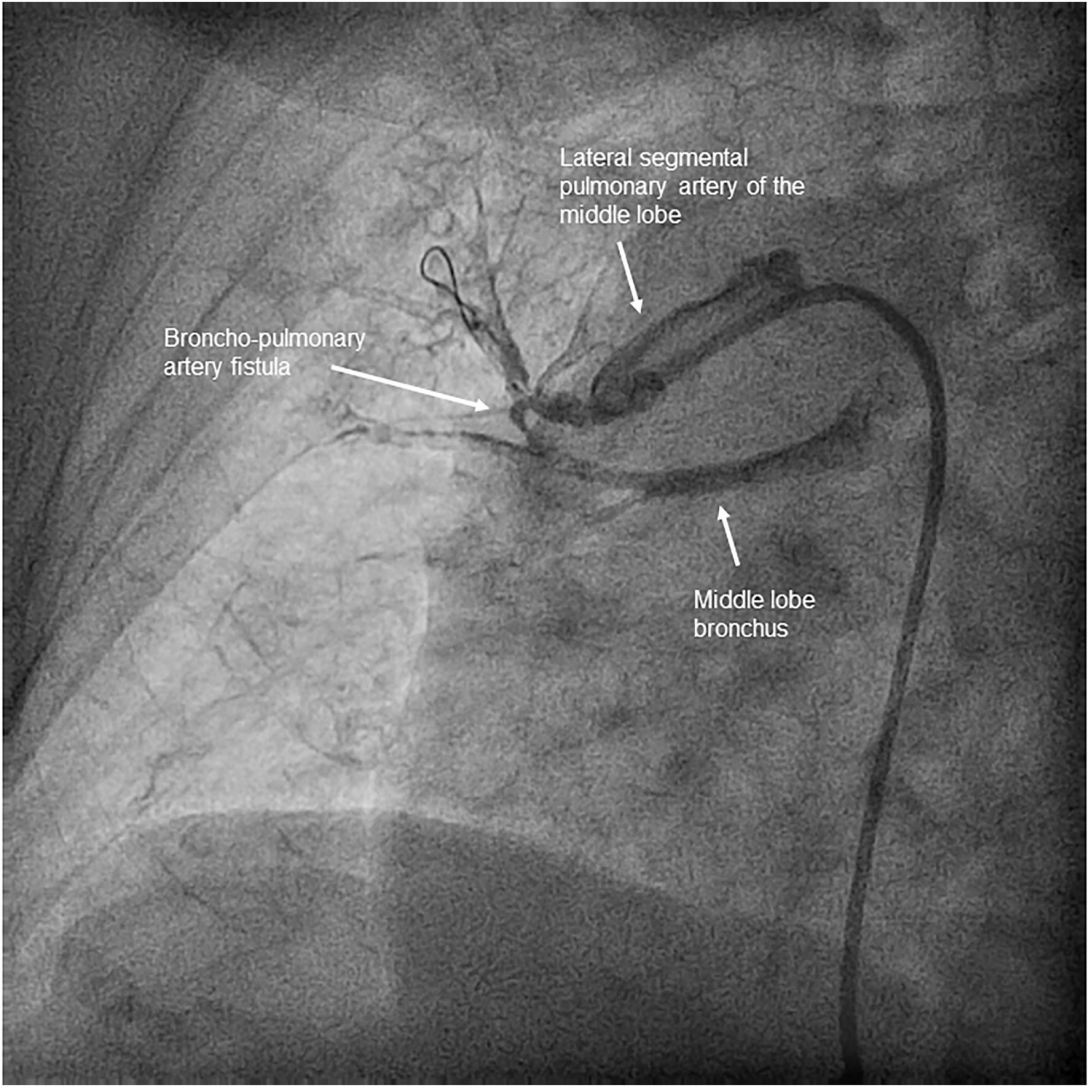
Bronchopulmonary artery fistula. Significant contrast leakage from one pulmonary artery branch of the middle lobe to the bronchus, without visible parenchyma extravasation, which appeared after advancing a 2.5 mm balloon without inflation through a subtotal stenosis.

**Figure 6:**
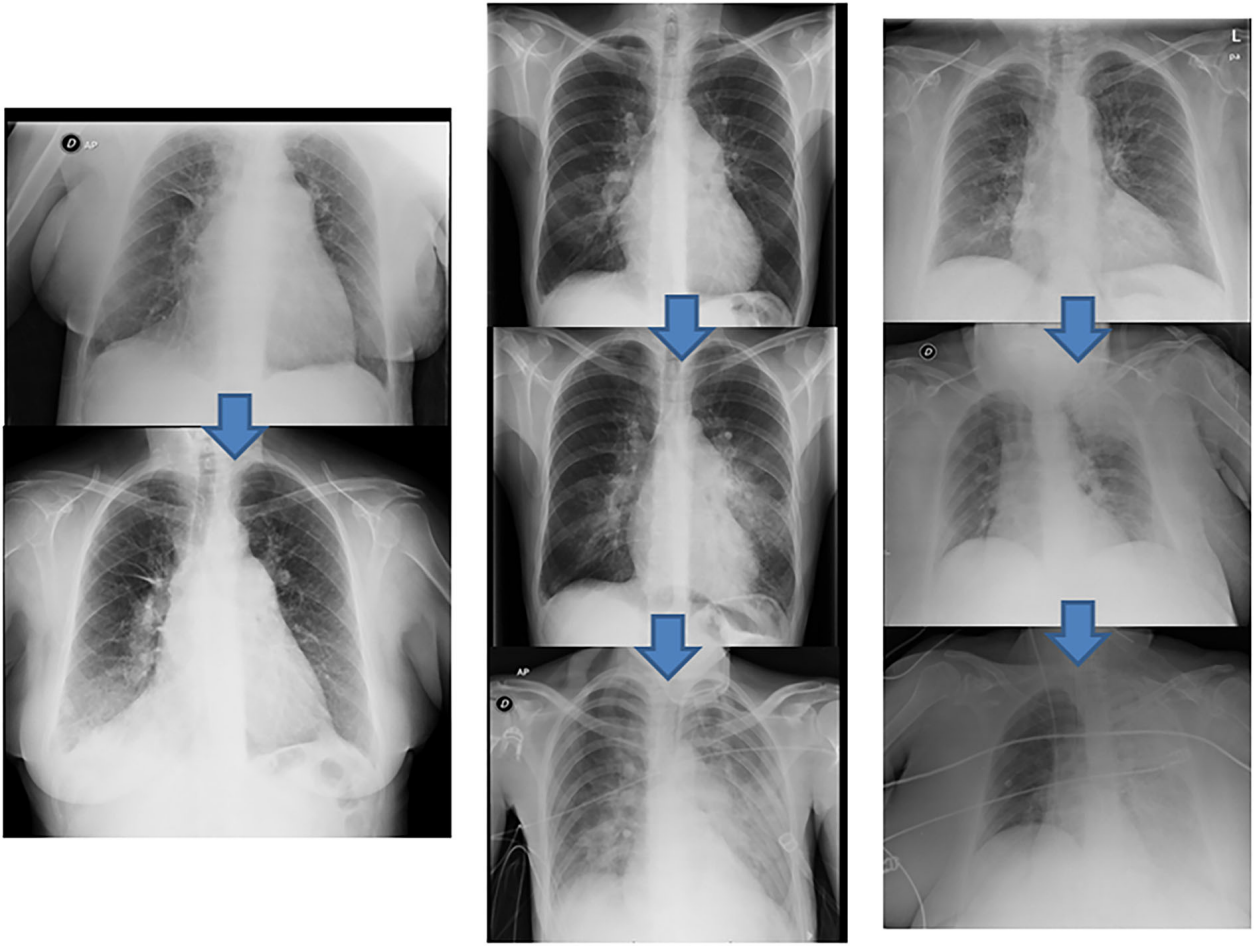
Radiologic pulmonary injury expression. Examples of different degrees of reperfusion edema according to their radiological expression. **(Left)** grade 3 reperfusion edema, infiltration in the right lower lobe (above pre-BPA baseline chest radiograph). **(Center)** grade 4 reperfusion edema, infiltration in the lingula extending to the contralateral lung (above pre-BPA baseline chest radiograph). **(Right)** grade 5 reperfusion edema, initial infiltration in the left upper lobe that eventually spreads to the entire left lung (above pre-BPA baseline chest radiograph). BPA, balloon pulmonary angioplasty.

Across all 116 procedures performed on 33 patients, the most common complication was mild hemoptysis, which occurred in 21.2% of the patients throughout the program and in 12.9% of the procedures. This complication was resolved in all patients by stopping the procedure, along with anticoagulation reversal and prolonged balloon inflation when necessary (Supplementary Videos 3–5). None of the patients with this complication required ventilatory or hemodynamic support. One patient underwent covered stent implantation due to a bronchopulmonary artery fistula (Figure 5). The pulmonary injury appeared in 3.4% of the procedures, of which 50% were grade 2 (Figure 6). No patient aged ≥70 years had a grade 5 pulmonary injury. One patient who was admitted to undergo several BPA procedures died 96 h after her first BPA session due to a hemorrhagic stroke while receiving LMWH at an anticoagulant dose (periprocedural mortality, 3%) (Table 5).

**Table 5:** All procedure-related complications in the cohort aged ≥70 years.

**Variables**	**Per-patient (*n =* 33)**	**Per-procedure (*n =* 116)**
Reperfusion edema %, (n)	12.1 (4)	3.4 (4)
• Grade 2	6.1 (2)	1.7 (2)
• Grade 3	3.0 (1)	0.9 (1)
• Grade 4	3.0 (1)	0.9 (1)
• Grade 5	0	0
Hemoptysis %, (n)	30.3 (10)	15.5 (18)
• Mild	21.2 (7)	12.9 (15)
• Severe	9.1 (3)	2.6 (3)
Access-site hematoma %, (n)	3.0 (1)	0.9 (1)
Access-site infection %, (n)	3.0 (1)	0.9 (1)
Pulmonary vascular dissection %, (n)	6.1 (2)	1.7 (2)
Pulmonary vascular perforation %, (n)	6.1 (2)	1.7 (2)
Contrast allergy %, (n)	12.1 (4)	3.4 (4)
Bronchopulmonary fistula %, (n)	3.0 (1)	0.9 (1)
Acute pulmonary embolism %, (n)	3.0 (1)	0.9 (1)
Acute renal insufficiency %, (n)	3.0 (1)	0.9 (1)
Periprocedural mortality %, (n)	3.0 (1)	

### Events during follow-up

**Figure 7:**
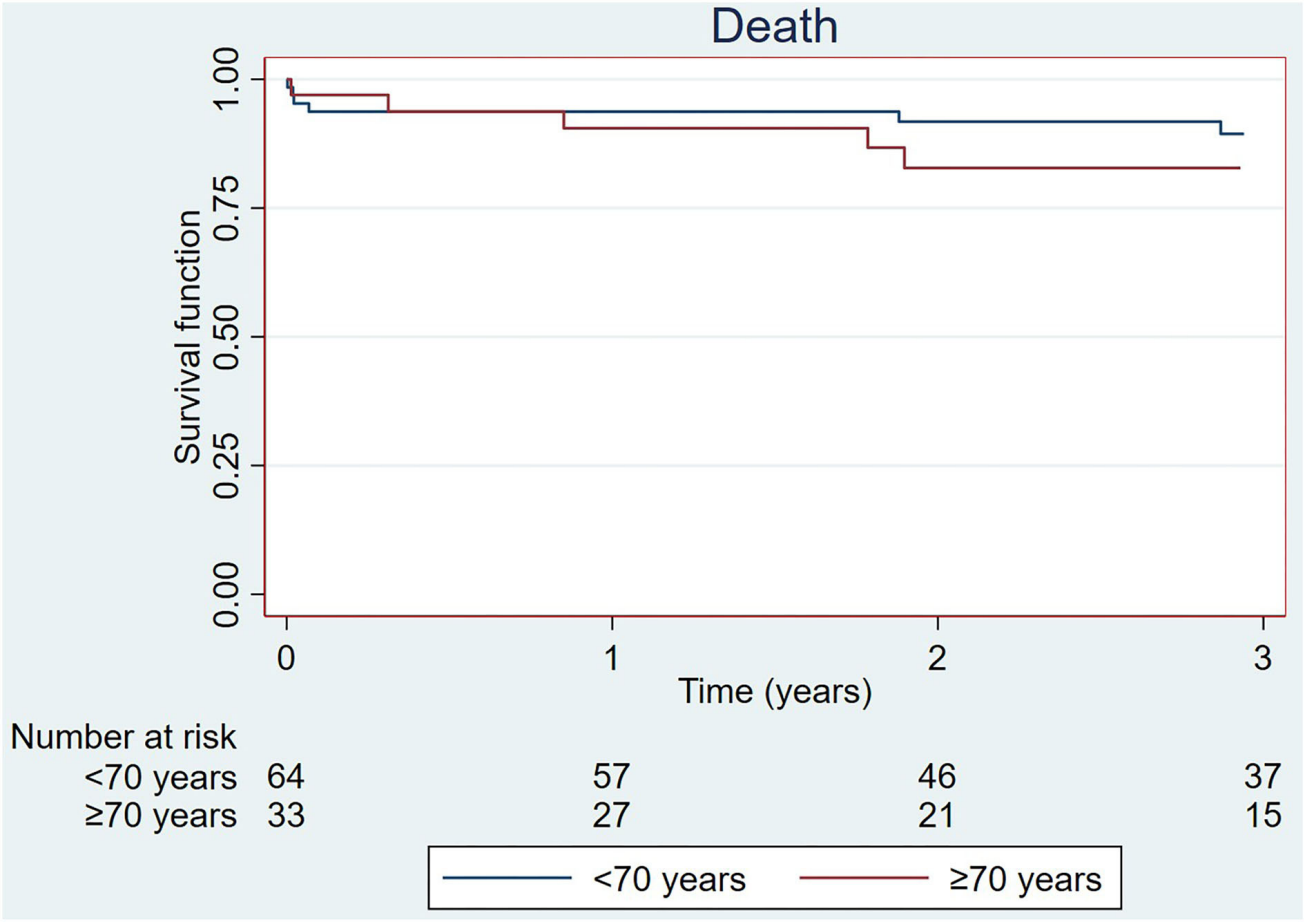
Survival of the cohort ≥70 years and that of the population < 70 years with finished BPA therapy treated at the same center during the same period. BPA, balloon pulmonary angioplasty.

The median follow-up period was 2.8 years (IQR: 1.7–3.8). Survival of the entire cohort of patients aged ≥70 years was 90.5 and 82.8% at 1 and 3 years, respectively. A comparison of the survival of the study group with that of the population aged < 70 years who had completed therapy of all lobes (*n* = 64) at our center at the time of analysis showed no significant differences between groups (Figure 7). In the group aged < 70 years, survival was 93.7 and 89.4% at 1 and 3 years, respectively (log-rank test; *p* = 0.63 for 1 year and *p* = 0.20 for 3 years). Among the patients aged ≥70 years, one died during the peri-procedure and eight patients died during follow-up. Among all the deaths, 66% were related to PH (Table 6). Three deaths occurred due to right heart failure progression in patients who had interrupted BPA therapy. One death occurred due to bronchoaspiration in a patient 5 years after completing BPA therapy. The remaining four deaths occurred in patients who were still undergoing BPA: two due to right heart failure, one due to an ischemic stroke, and one due to leukemia. Data for each event can be found in Table 3.

**Table 6:** Events during follow-up.

**Follow-up events (*****n*** = **33)**		
**Events**	**Incidence (%)**	**Incidence rate (100 people-year)**
Death	9 (27.3)	9,9
**Cause of death**		
Related to PH	6 (66.6)	
Not related to PH	3 (33.3)	

PH, Pulmonary hypertension.

## Discussion

In the present study, we examined the tolerance, effectiveness, complications, and outcomes of BPA in patients aged ≥70 years. The main findings of our work are as follows: (1) elderly patients benefited from BPA, with functional class and hemodynamic improvements, reductions in biomarker levels, and an important reduction in PH-targeting medical therapy requirements; (2) the rate of severe complications related to the BPA procedure in this population is low; (3) in-hospital mortality in this population is also low; (4) the all-cause mortality rate of elderly people at 1 and 3 years after BPA is low and comparable to that of patients aged < 70 years treated at the same center during the same period; (5) mortality at the mid-term follow-up is mainly driven by right heart failure progression; and (6) a high percentage of these elderly patients do not undergo complete therapy of all lobes, mainly due to complications or absence of improvement.

BPA is an emerging therapeutic modality for CTEPH patients disqualified from PEA. Elderly patients can be too fragile for PEA. Thus, BPA, a less invasive therapy, may be an attractive alternative therapeutic strategy for these patients, provided the results in this population are good. However, the literature contains little information about the safety, procedure tolerance, effectiveness, and outcomes of BPA in elderly patients. An initial study reported that CTEPH patients aged over 65 years benefited from BPA, with similar efficacy and safety to that observed in younger patients (9). However, that study included only 31 patients and chose a relatively young age for identifying elderly patients (65 years). Two more recent studies in elderly patients have confirmed the effectiveness of BPA, although they included only 10 patients aged ≥75 years (9) and eight patients aged ≥70 years (10). A French study that compared BPA and PEA results in patients aged ≥80 years also showed good results in the 21 patients who underwent BPA (11). Our work, which includes a larger number of elderly patients than the previous series, provides information regarding tolerance, effectiveness, and safety in this population.

### Functional class, procedure tolerance, and futility of the therapy in elderly patients

Although the main goals of PEA or BPA are to improve survival, quality of life, and functional class, survival improvement in elderly patients is often less prioritized, with improvements in quality of life and functional class being the main objectives of these therapies. In the BPA series worldwide, although hemodynamic parameters improved substantially after BPA, many patients still experienced symptoms corresponding to the World Health Organization functional class (WHO-FC) ≥ II at follow-up, even after the completion of BPA therapy of all amenable lesions. This could be more marked in elderly people with CTEPH, who are older than those with group 1 PH and have many comorbidities (1, 12). Moreover, information regarding BPA procedure tolerance in this patient population is quite limited. Nevertheless, it is easy to understand that elderly patients can poorly withstand extended procedures on a catheterization table with their arms raised. Furthermore, futility in elderly patients is another potential issue, since comorbidities and physical deconditioning may partly explain their functional class, which would continue to remain suboptimal after BPA therapy as a result of these comorbidities. The female predominance in our cohort indicated a higher degree of fragility and possible futility of BPA therapy since older women are often weaker and more fragile (13). Taking these factors into consideration, we think that a significant functional improvement defined by WHO FC 1 or 2 could be a potentially sufficient aim, especially in a population of elderly patients like ours. In our cohort, after BPA therapy, 73% of the patients were in WHO FC I or II, while the corresponding proportion before beginning BPA therapy was only 21%. This improvement is similar to the findings reported in other series of elderly patients such as the study by Roik et al., in which all patients improved to functional class I or II (14), or the French study, which reported that most patients reached functional class I or II without providing the actual percentages (11). In our series, the BPA program was interrupted in 14 patients (42%). Despite this high percentage of BPA program interruption, our series shows an overall benefit, with a significant functional class improvement and an increase of 25 m in the 6MWD. Nevertheless, the high percentage of program interruption needs to be minimized. In this regard, a better selection of BPA candidates is mandatory to enhance therapy performance. Exclusion of patients who are extremely frail, have severe osteoarticular mobility problems, or show cardiovascular and respiratory comorbidities would help to achieve this goal.

### Hemodynamic and biomarker improvement

Significant improvements in hemodynamic parameters and biomarkers have been described in all BPA series (4, 5), including those that recruited elderly patients. Thus, in the study by Roik et al., BPA therapy resulted in the normalization of mPAP (< 25 mmHg) in six of the 10 patients aged ≥75 years (14). Likewise, in the study by Yamagata et al., mPAP changed from 43 ± 6.8 to 19.8 ± 3.1 (*p* < 0.001) (10). PVR was reduced by 5 W.U. in both series, although only the first showed CO improvement. Although the corresponding findings in our series were statistically significant, the magnitude of the hemodynamic benefit was lower in terms of mPAP, which reduced from 39.2 ± 9.3 to 32.8 ± 8.8 mmHg, or PVR, which reduced from 6.7 ± 3.1 to 4.4 ± 2.0 W.U. However, the cardiac index improved significantly in our patients, from 2.5 ± 0.6 to 2.8 ± 0.7 (*p* = 0.04). The less impressive improvement in mPAP and PVR in our series are partially related to the high rate of interruption of BPA therapy for the previously explained reasons. The reductions in mPAP and PVR among patients who completed the therapy were 23 and 38%, respectively, while the corresponding values in the entire group were 16 and 34%, respectively. Our results also showed improvements in biomarker levels, with a significant reduction in the level of NT-proBNP similar to that shown in the series by Roik et al. (14). In contrast, the series by Yamagata showed no benefit in biomarkers or cardiac output, probably due to the small sample size (10).

### Complications

According to the literature, BPA seems to be a safe procedure in elderly patients. Some studies have reported a higher rate of mild complications than in non-elderly patients, such as transient hemoptysis, without fatal complications (9, 10). In the French study, which compared complications in elderly patients who underwent BPA or PEA to those in younger patients, the two groups showed no differences except in the incidence of contrast-induced nephropathy (8.4% in the elderly group vs. 2.6% in the younger group). However, no periprocedural deaths due to BPA or PEA were reported in that cohort (11). In comparison with these studies, our cohort showed a higher incidence of pulmonary injury (12% of the patients) and intra-procedural transient hemosputum/hemoptysis (15% of the procedures). Although the rate of fatal complications in our series was low, one patient died 4 days after her first BPA procedure due to a hemorrhagic stroke and one patient developed a bronchopulmonary artery fistula during her fifth BPA session. The second patient was the only one who required mechanical ventilation due to severe hemoptysis (15). This complication was solved by covered stent implantation. The patient refused to continue with the therapy after this complication. None of the patients required extracorporeal membrane oxygenation. The incidence of contrast nephropathy in patients aged over 70 years in our series was lower than reported (3%), while it was 6% in patients aged under 70 years treated at our center.

### PH-targeting medical therapy requirements

Our center is one of the two reference centers accredited by the National Health System of our country to perform the BPA therapy, and the one with the largest number of patients. Thus, our waiting list for BPA therapy is long. This forced us to optimize the use of this therapy, giving priority to patients with a poor functional or hemodynamic conditions instead of completing the therapy in patients who had already experienced significant improvement after undergoing two or three BPA sessions. Furthermore, the COVID-19 pandemic has lengthened the duration of BPA therapy due to periodic admission restrictions over the last 3 years. Therefore, the time taken to complete the therapy for all lobes was nearly 1 year in our series. In addition, we pre-treated all patients with an intermediate-high risk hemodynamic or clinical profile to reduce procedural complications. For these reasons, most of our patients received PH-targeting drugs for the duration of the BPA therapy, with the aim of alleviating symptoms and improving the patient's risk profile. The reduction in the requirement of PH-targeting drugs is a well-known benefit of BPA therapy, and this was observed in our series as well, with a mean reduction of 0.39 in the number of PH-targeting drugs (95% CI, 0.11–0.67; *p* = 0.01) after completion of BPA therapy. Thus, at follow-up, 50% of the patients who received two drugs at baseline could stop taking at least one drug and 27% of the cohort could stop taking all PH-targeting drugs at the end of therapy, while only 6% of patients had no treatment at baseline.

### Survival and event-free survival during follow-up

Several series have shown global favorable medium-term survival after BPA (3, 16, 17). However, only a few series have analyzed survival after BPA in elderly patients. The published findings suggest that survival in this patient population is not worse than that in younger patients. In the series reported by Roik et al. (14) all patients were alive without severe complications during follow-up, with a median follow-up period of 553 days (range, 81–748 days). Two patients were re-hospitalized in the initial phase of BPA therapy due to decompensation of right ventricular heart failure requiring transient intravenous diuretic therapy (14). In the series by Yamagata et al., no periprocedural deaths were recorded, although the authors provided no information about the medium- or long-term survival (10). In the study by Wiedenroth et al. (17), a contemporary series that analyzed survival after PEA at an expert PEA center, the in-hospital mortality rate was 2.1% in patients aged >70 years, with 6.3% overall mortality at 1 year. Upon evaluating the overall mortality after PEA in younger patients, they found lower 1-year mortality rates of 1.1% in patients aged ≤ 50 years and 3.2% in those aged >50– ≤ 70 years, although the differences were not statistically significant. Similarly, in our series, the outcomes after BPA were comparable between the elderly patients and the younger cohort who had completed BPA therapy at our center during the same period, although they were slightly worse in the elderly population. Survival was 90.5 and 82.8% at 1 and 3 years, respectively, in the group aged ≥70 years, while in the group aged < 70 years, the corresponding values were 93.7 and 89.4%. The main cause of mortality during follow-up in elderly people was right failure progression, which accounted for three deaths in the group with interrupted therapy and two deaths in patients who were under an active BPA program. Most patients who died due to right heart failure progression at follow-up were receiving two or three PH-targeting drugs, indicating that these patients had very advanced forms of the disease with significant degrees of pulmonary vasculopathy. Only one of the patients who completed BPA therapy died during follow-up, at 5 years after BPA therapy completion, and of causes unrelated to PH. These findings, which place survival after BPA at levels similar to those after PEA surgery, reinforce the role of BPA in elderly patients not suitable for PEA.

### Limitations

This was a single-center series with a small number of patients. Although we obtained 100% of the data for the hemodynamic parameters, number of drugs, and functional classification, mobility restrictions in different regions in our country, and patients' reluctance to come to the hospital for consultation during the COVID-19 pandemic meant that complete data for pre- and post-BPA NT-proBNP and 6MWD values were obtained in only 72.7 and 66.7% of the patients, respectively. One aspect that would be especially interesting in the elderly would be the findings of a quality-of-life survey, which we did not conduct. Nevertheless, we think that the improvement in functional class, increase in the distance walked, and decrease in the number of drugs needed reflect an improvement in the patients' quality of life.

## Conclusion

Our findings obtained, to our knowledge, the longest series of elderly patients treated with angioplasty and showed that BPA is effective and quite safe in patients aged ≥70 years. It significantly improves the functional class as well as hemodynamic and biomarker parameters and allows physicians to reduce PH-targeted medical therapy after completing the therapy for all lobes. These successful outcomes were achieved even though a substantial proportion of patients interrupted therapy before completing treatment for all lobes. Moreover, the rate of procedure-related complications and periprocedural mortality was low and survival at 1 and 3 years was good, comparable to that in younger patients and elderly non-surgical patients who underwent PEA. Mid-term mortality was mainly driven by right heart failure progression.

## Data availability statement

The raw data supporting the conclusions of this article will be made available by the authors, without undue reservation.

## Ethics statement

The studies involving human participants were reviewed and approved by Comité ético del Hospital Universitario 12 de Octubre de Madrid. The patients/participants provided their written informed consent to participate in this study.

## Author contributions

MV, NM, and PE designed, drafted, revisited critically and made the final approval of the manuscript. AAG-T, FS, AC-U, SH, WH, RA, SA, ML-G, YR, and FA revisited critically and made the final approval of the manuscript. All authors contributed to the article and approved the submitted version.

## Conflict of interest

The authors declare that the research was conducted in the absence of any commercial or financial relationships that could be construed as a potential conflict of interest.

## Publisher's note

All claims expressed in this article are solely those of the authors and do not necessarily represent those of their affiliated organizations, or those of the publisher, the editors and the reviewers. Any product that may be evaluated in this article, or claim that may be made by its manufacturer, is not guaranteed or endorsed by the publisher.

## Supplementary material

Supplementary Videos 1 Incomplete distal perfusion due to organized material within a segmental lower lobe.

Supplementary Videos 2 Signs of effective lesions balloon dilation. The distal flow improves, contrast uptake in the lung tissue distal to the treated area increases, and the pulmonary venous return to the left atrium improves. Post-balloon dilation.

Supplementary Videos 3 Total occlusion of the cranial lingula branch at middle level.

Supplementary Videos 4 Distal guidewire perforation of the cranial lingula branch during balloon pulmonary angioplasty.

Supplementary Videos 5 Prolonged transient balloon inflation in the cranial lingula branch to stop extravasation and hemoptysis.
